# Influence of Silicon Additives on Tribological and Rheological Test Results for Vegetable Lubricants

**DOI:** 10.3390/ma16186245

**Published:** 2023-09-16

**Authors:** Rafal Kozdrach, Jolanta Drabik, Marian Szczerek

**Affiliations:** 1 Department of Bioeconomy and Ecoinnovation, Lukasiewicz Research Network—Institute for Sustainable Technologies, 26-600 Radom, Poland; 2 Tribology Department, Lukasiewicz Research Network—Institute for Sustainable Technologies, 26-600 Radom, Poland

**Keywords:** vegetable lubricants, antiwear properties, antiscuffing properties, dynamic viscosity, viscosity curves, flow curves, yield point, mean square displacement, G′ and G″ modules

## Abstract

This paper describes an investigation of the effects of silicone-containing additives on the tribological and rheological properties of various lubricant blends. Aerosil^®^ and layered silicate were used to modify lubricants containing rapeseed, linseed and soy oil that were thickened with soap thickener. Tribological tests were carried out using a four-ball concentric contact tester. On the basis of the data obtained from the tribological studies of the selected lubricant blends, it was concluded that the addition of amorphous silica increased the anti-seizure and anti-wear properties of the tested lubricants. The addition of montmorillonite caused a significant increase in the values of the individual parameters determining the level of lubricating properties of the tested lubricants in comparison with the lubricants modified with the silica additive. Based on the results of the rheological tests of the studied lubricants, it was found that the applied additives caused a change in the dynamic viscosity and chemical structure of the tested lubricants, expressed by a change in the values of the G′ and G″ indices. The main finding of this manuscript was to demonstrate that the use of montmorillonite and aerosil additives improves the functional properties of vegetable-based plastic lubricants. The performance of tribological and rheological tests is of great scientific importance, as it provides an insight into the interaction of siliceous additives with the results of tribological tests on vegetable-oil-based greases. These findings make it possible to determine the behaviour of the lubricant under load and add to the knowledge of vegetable greases.

## 1. Introduction

The behavior of greases depends on their formulation and manufacturing technology and is influenced by the choice of additives [1,2,3,4]. The most common additive packages that modify lubricants include, for example, antioxidants (which increase the stability of the lubricant against oxidation), anti-wear and anti-scuffing additives (which improve the tribological properties of the lubricant), anti-corrosion additives (which reduce the aggressiveness of the lubricant against metals), adhesion additives (which improve the ability of the lubricant to adhere to equipment parts) and rheological additives (which improve the ability of the lubricant to withstand changes in viscosity). It is not only the presence of the additive that influences the useful properties of the lubricant, but also the way in which it is incorporated into the structure of the lubricant. The incorporation of additives into lubricants poses a number of technical problems, as the particles of the additive are adsorbed on the surface of the dispersed phase, which can reduce the effectiveness of the ingredient and the durability of the grease [5,6,7,8,9].

Appropriate, specially selected additives should be added to lubricants in quantities that determine the effectiveness of their properties. Lubricants can be easily mixed with solid lubricant additives that can reduce frictional forces, increase the stability of the tribological system against loads and wear, and modify the rheological properties of the lubricant [10,11,12,13,14]. Under severe operating conditions, these additives increase the efficiency of the grease due to their ability to resist chemical attacks and withstand exposure to high temperatures. The most commonly used additives of this type are graphite, molybdenum disulphide, polytetrafluoroethylene, copper and chloroparaffins [15,16,17,18,19,20].

**Figure 1:**
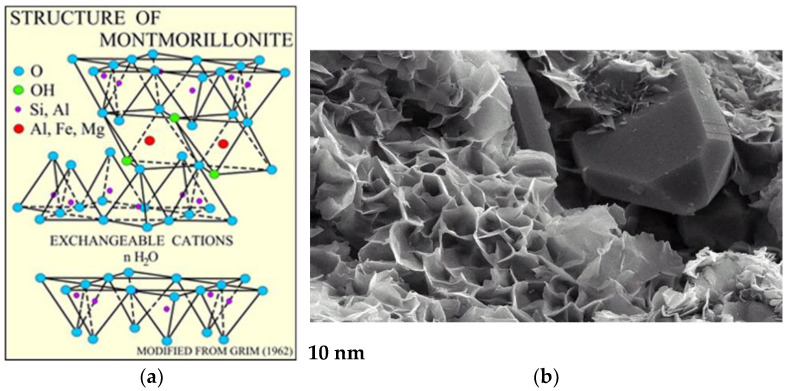
The chemical structure of layered silicate (**a**) and SEM micrograph of the microstructure of layered silicate (zoom 4000×) (**b**) [31,32].

Modern science has applied its innovation to nanotechnology, in which structures with at least one size below 100 nm are used [21,22,23,24]. The incorporation of nanoadditives into the grease matrix significantly improves its anti-seize and anti-wear properties as well as its rheological behaviour, which determines the useful properties of the lubricant. These properties explain the great interest in innovative ceramic nanoadditives. The negative aspects of nanoadditives are their high cost, limited availability and difficulties in achieving the appropriate degree of dispersion in the lubricant structure. The proportion of ceramic nanoadditives introduced into lubricant compositions at a concentration of 7–10% is sufficient to meet the particularly high demands placed on lubricants [25,26,27,28]. A particularly important role is played by multilayer nanosilicates, the most commonly used of which is montmorillonite with the formula M_x_(Al_4−x_Mg_x_)Si_8_O_20_(OH)_4_ and a particle size of 100–150 nm [29,30]. They are composed of 2:1 three-layer packs with an octahedral layer sandwiched between two tetrahedral layers (Figure 1) [31,32]. The octahedral layer is composed of aluminium or magnesium oxide and is connected to two external silicon (tetrahedral) layers by shared oxygen atoms. Modified with quaternary ammonium salts, it becomes hydrophobic and organophilic, allowing it to absorb the same amount of organic liquids, such as oils. Modified montmorillonite is compatible with greases and is used as an additive to modify their lubricating and rheological properties. Organic hydrophobic agents are chemically bonded to the surface of the montmorillonite, which allows the montmorillonite to be permanently bonded to lubricants. In addition, cation exchange increases the interlayer distance from approximately 1 nm for natural montmorillonite to 2–3 nm for montmorillonite modified with organic compounds [33,34,35].

**Figure 2:**
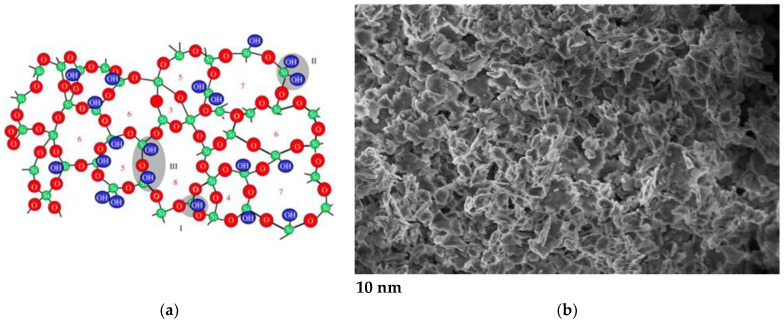
The chemical structure of Aerosil (**a**) and SEM micrograph of the microstructure of Aerosil (scale bar 4000×) (**b**) [43,44]. The numbers 3–8 are the number of groups of (Si–O–) and I–III are the numbers of Si–OH groups.

The second-most commonly used ceramic nanoadditive after phyllosilicates is silica, i.e., silicon (IV) oxide, which produces extensive three-dimensional structures. The improvement in properties is attributed to the large specific surface area and very good interfacial interactions [36,37,38,39]. Methylated silica is a hydrophobic form of silica modified with dimethyldichlorosilane (Figure 2). This treatment significantly reduces the phenomenon of silica grain agglomeration, allowing for easier and more effective introduction of the dispersed phase particles into the lubricants under mild mixing requirements. The rheological properties are the main determinants of the functional properties of the grease, which determine its application possibilities [40,41,42]. Colloidal silica forms complex spatial structures in which each silicon atom is bonded to four oxygen atoms and each oxygen atom is bonded to two silicon atoms. There are many defects in the crystal structure, and the silica surface also contains hydrogen atoms or hydroxyl groups. It is a material with a relatively high melting and boiling point, it is highly resistant to chemicals and it is non-toxic, which is essential in the formulation of biodegradable greases. It is used as a rheological additive and as an excellent thickener, thixotrope and anti-sedimentation agent. Due to its polar nature, it is easily associated with oil molecules by van der Waals forces [43,44].

Many scientific publications can be found for vegetable lubricants modified with montmorillonite and amorphous silica Aerosil. These publications mostly use tribological tests based on the four-ball method [29,45,46], tribological tests using a tribological attachment in a rotational rheometer [47,48] or a Falex tribotester using a ball–plate contact tester [49]. A ball-on-disk configuration [50] and a pin-on-disk tribometer have been used to simulate the boundary lubrication condition for hydrodynamic plain bearings [51].

The novelty of this paper is the use of additives in the form of montmorillonite and Aerosil and their positive effects on the rheological and tribological properties of vegetable-oil-based lubricants. The aim of this paper was to investigate the effect of montmorillonite and amorphous silica on the tribological and rheological properties of vegetable greases.

## 2. Materials and Methods

A new generation of prototype lubricant formulations was created based on non-toxic components in base oils and thickeners. Based on the results of physico-chemical, oxidation, rheological and tribological tests, oil bases were selected to formulate the lubricant compositions that were used in further experiments. Lubricants based on rapeseed, linseed and soybean oils were used for the tribological and rheological tests (Table 1 and Table 2) [29,52,53,54]. Montmorillonite was purchased from Sigma-Aldrich (Saint Louis, MO, USA), Aerosil 300 from OQEMA (Ozorków, Poland), lithium stearate from Linegal Chemicals and vegetable oils from iZielnik (linseed oil, Białystok, Poland), Ekoflos (rapeseed oil) and Agnex (sunflower oil, Białystok, Poland).

Lithium stearate was used as a thickener for the greases tested. The grease formulations prepared in this way were then modified with ceramic nanoadditives in the form of aerosil and layered silicate (Table 3) [10,11,12,13,29]. 

The tests were carried out with formulations containing between 1 and 8% of each additive. The above additives were incorporated into the base formulation of each lubricant at a level of 5% *w*/*w*. The lubricant formulations were prepared by incorporating a thickener in the form of lithium stearate into the vegetable oil base. The introduced thickener was mixed with the vegetable oil base for 40 min while heating from 20 °C to 180 °C with simultaneous homogenisation at a spindle speed of 18,000 rpm. The additives used were incorporated into the lubricant compositions for 15 min with simultaneous mixing at a speed of 20,000 rpm at 25 °C. The lubricants produced using this method were then labelled with the following symbols: A (lubricant based on rapeseed oil), A1 (grease A modified with silica), A2 (grease A modified with layered silicate), B (grease based on linseed oil), B1 (grease B modified with silica), B2 (grease B modified with layered silicate), C (grease based on soybean oil), C1 (grease C modified with silica) and C2 (grease C modified with layered silicate). In the initial stages of the study, the amount of additive to be incorporated into the lubricant mixture was investigated.

The tribological behavior of the lubricants was evaluated using a T-02 four-ball tester.

The lubricating properties were measured by determining the limiting wear load (G_oz/40_), welding load (P_z_), scuffing load (P_t_), limiting scuffing load (P_oz_) and limiting seizure pressure (p_oz_) [61,66,67,68]. The samples of the experimental elements were steel balls with Ø 12.7 mm placed in a steel type bearing LH 15. The hardness of the balls was 60–65 HRC, and their roughness was Ra = 0.32 µm. Table 4 shows the chemical components of the steel balls. 

The wear limit load (G_oz/40_) was determined by applying a tribological force of 392.4 N for the entire test duration of 3600 s. The rotational speed of the ball was set to 500 rpm in accordance with the WTWT-94/MPS-025 procedure [60]. The welding load was evaluated according to PN-76/C-04147. This test was performed in a four-ball tester for 10 s in the presence of the grease under gradually increasing loads until the balls were welded. The measurement of the lubricating behavior during scuffing action (i.e., with the load continuously increased during the tests) was carried out according to the method described by tribologists.

The test was carried out with a gradually increasing force from 0 to 7200 N (ramp 409 N/s) over a period of 18 s at a speed of 500 rpm. The point at which the frictional load abruptly increases is known as the scuffing load P_t_. The test was continued until the friction value reached 10 Nm or the full load of the instrument, which was set at 7200 N. This moment was considered to be the maximum scuffing load P_oz_. The value for each individual measurement was taken as the arithmetic mean of at least three independent tests with a maximum difference of 10%. The Q-Dixon test with a 95% confidence interval was used to statistically process the data.

The limiting wear load is a criterion for the anti-wear performance of the grease formulation. The calculation of this value is based on Equation (1):

(1) $$G_{oz}=\frac{P_{n}}{{d_{oz}}^{2}}\times 0.52$$

where P_n_ is the friction node load equal to 392.4 N and d_oz_ is the size of the diathesis created on the steel plates used in the test.

The limiting pressure of seizure is a measure of the antiscuffing properties of lubricants under scuffing conditions. The estimation of this parameter is based on a calculation according to the Formula (2):

(2) $$p_{oz}=\frac{P_{oz}}{d{oz}^{2}}\times 0.52$$

where P_oz_ is the limiting load of scuffing [N] and d_oz_ is the scar diameter formed on the steel balls used in the tests. 

An electronic microscope was used to determine the diameter of the wear scar on the steel balls tested. The data were used to calculate the values of G_oz/40_ and P_oz_ or to evaluate the anti-wear and anti-scuffing properties of lubricants subjected to tribological studies [66,67,68,69,70,71,72,73,74].

An Anton Paar MCR-101 rotational rheometer with an air bearing was used to determine the rheological properties of the lubricants tested. The tests were carried out using a cone–plate geometry measuring system. The measuring system consisted of a rotating cone with a diameter of 2 cm rotating at a given speed, a Peltier element on which the sample to be tested was placed at an angle of 2° to the rotating cone, and a sensor to measure the specific rheological parameters.

The tests were performed at a temperature of 20 °C. The shear rate range over which the tests were carried out (0–100 s^−1^) and the measuring intervals at which the data were collected (number of measuring points, total measuring time and frequency of data collection) were then determined. For the viscosity curves, the temperature range was −10–100 °C and the shear rate was 1000 s^−1^ [11,12,14]. For the flow curves, a logarithmic curve was used in order to collect more points at the beginning of the range, which is crucial for determining the minimum flow limit.

Four rheological models were used to describe the flow curves of the lubricant compositions tested: Bingham, Casson, Herschel–Bulkley and Tscheuschner [75,76,77,78,79,80,81,82].

The Casson model describes the flow curves of non-linear plastic viscous fluids as follows (3):

(3) $$\tau_{1/2}={\tau_{0}}^{1/2}+(\eta_{\infty }\times \gamma )$$

where:

τ—shear stress [Pa]

τ_0_—yield point [Pa]

η_∞_—the structural viscosity of the grease [Pa·s]

γ—shear rate [1/s].

The Bingham model describes the determination of shear stress from shear rate in a linear way (4):

(4) $$\tau =\tau_{0}+\eta_{p}\times \gamma$$

where:

*τ*—shear stress [Pa],

*η_p_*—the structural viscosity of the grease [Pa·s],

*τ*_0—_yield point [Pa],

*γ*—shear rate [s*^−^*^1^].

The Herschel–Bulkley model is the simplest model to describe the flow curves of non-linear plastic–viscous fluids (5):

(5) $$\tau =\tau_{0}+k\times \gamma_{n}$$

where:

*τ*—shear stress [Pa],

*τ*_0_—yield point [Pa],

*k*—consistency coefficient [Pa·s^2^],

*γ*—shear rate [s^−1^],

*n*—the flow index [-]

Tscheuschner’s rheological model describes the flow curve of non-linear viscoelastic fluids (6):

(6) $$\tau =\gamma (\eta_{\infty }+\frac{\tau_{0}}{\gamma +\frac{{(\frac{\eta_{b}}{\gamma })}^{n}}{\gamma_{b}}})$$

where:

*τ*—shear stress [Pa],

*τ*_0_—yield point [Pa],

*γ*—shear rate [s^−1^],

*n*—the flow index [-],

η_∞_—the structural viscosity of the grease [Pa·s], 

*γ_b_*—shear rate [s^−1^],

*n_b_*—the flow index [-].

The study of the rheological properties of the lubricant mixture investigated was carried out using a DWS RheoLab instrument, an optical rheometer manufactured by the Swiss company LS Instruments AG (Fribourg, Switzerland). This instrument uses diffusing wave spectroscopy (DWS) to measure the rheological behaviour of materials, both solid and liquid, such as suspensions, dispersions, slurries, and oils [13,47,83,84]. The operating principle of the diffuse spectrometer is based on the idea that light propagation in optically opaque samples can be considered as a diffusion phenomenon. This instrument allows for micro-rheological studies of samples under conditions of static internal molecular motion in a wide range of frequencies, viscosities and elasticities. The optical spectrometer allows experiments to be carried out in two modes, transmission and backscattering. In transmission mode, the scattered intensity is determined after propagation through the material, and variations in intensity are correlated using the correlation intensity function (ICF). In the backscattering regime, the light returning to the incident beam is recovered and its oscillations are determined [85,86,87]. Before starting the experiments, the instrument was calibrated with a 222 nm diameter polystyrene emulsion in aqueous solution. The refractive index of the dispersion phase of each lubricant mixture, the duration of the test, the temperature and the dimensions of the spectrometer cell into which the mixture was introduced were then defined. The lubricants were prepared by introducing aTiO_2_ particles with a diameter of 360 nm into their matrix. The material was then homogeneously mixed and introduced into a spectrometer cell. The rheological measurements were carried out using a cuvette with an optical path length of 1 mm [88,89,90,91]. The duration of the test was 90 s. In the rheological studies, the mean square displacement (MSD) of the sample, the comprehensive viscosity, the complex index G*, the elastic index G′ and the viscous index G″ in steady-state mode were obtained as a function of frequency, and the correlation function as a function of delay time was obtained. The rheological tests were carried out at a temperature of 20 °C. Based on the evaluation of the measured rheological values, the variation of the viscoelastic properties of the studied lubricant blends was analyzed [92,93,94,95,96].

Raman spectroscopy provides information on the chemical structure of materials subjected to temperature and mechanical stress during processing and allows structural changes to be monitored over time. Observation of changes in the chemical structure of materials allows a comprehensive assessment of the influence of process conditions on the state of their structure and provides a comprehensive characterization of the integrity of the systems under investigation at the microscale [97,98,99,100,101]. A Raman spectrometer NRS 5100 (Jasco Corporation, Tokyo, Japan) with a scanning laser at a 532.12 nm wavelength and a CCD detector was used to investigate the structural changes in the chemical composition of the lubricants after the tribological studies. The specifications of the instrument were as follows: grating 2400 lines/mm, laser power 3.6 mW, digital resolution 3000 μm, spectrum 3700 ÷ 200 cm^−1^, resolution 2.1 cm^−1^, zoom lens 20× and exposure time 40 s. The samples were made of steel after the tribological tests [102,103,104,105].

## 3. Results and Discussion

### 3.1. Tribological Test Results

**Figure 3:**
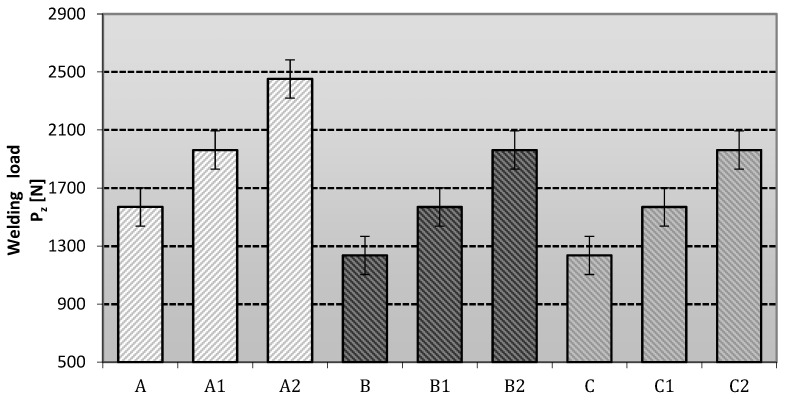
Welding load of friction node with lubricants with different vegetable dispersion phases and modified by silicon nanoadditives. A–C: designation of the tested grease.

The data from the tribological studies (anti-scuffing and anti-wear properties) of lubricants containing different vegetable-base oils modified with ceramic nanoadditives are presented below. The welding load P_z_ was determined for selected lubricant compositions. The results obtained are shown in Figure 3.

The antiscuffing characteristics with gradually increasing loads on the friction node for the tested lubricants depend on the type of base oil and the type of modifying additive used, which significantly affects the lubricating properties of the prepared grease compositions and the stability of the tribological film (Figure 3). The most favorable anti-scuffing protection is characteristic of greases based on rapeseed oil (A2) modified with one of the layered silicates, i.e., montmorillonite. The lubricating compositions based on linseed and soybean oil and modified with the above-mentioned nanoadditive (B2 and C2) did not show such favourable changes in the anti-scuffing properties as the A2 compositions, although an improvement in the properties is noticeable in relation to the base composition without the modifying additive.

On the other hand, the lubricating compositions modified with amorphous silica (Aerosil) showed slightly lower anti-scuffing properties than the greases modified with layered silicates, regardless of the vegetable base oil used.

The most favourable effect of amorphous silica on the improvement of the anti-scuffing properties compared to the base compositions can be observed for lubricants based on rapeseed oil (A1), whereas the effect of this modifier is lower for the compositions based on linseed oil (B1) and soybean oil (C1). Amorphous silica is a modifier that is less effective than montmorillonite—a representative of the layered silicates. Aerosil and montmorillonite as additives improved the anti-seize protection of the lubricants studied satisfactorily, but the more effective anti-seize effect was represented by the phyllosilicate.

**Figure 4:**
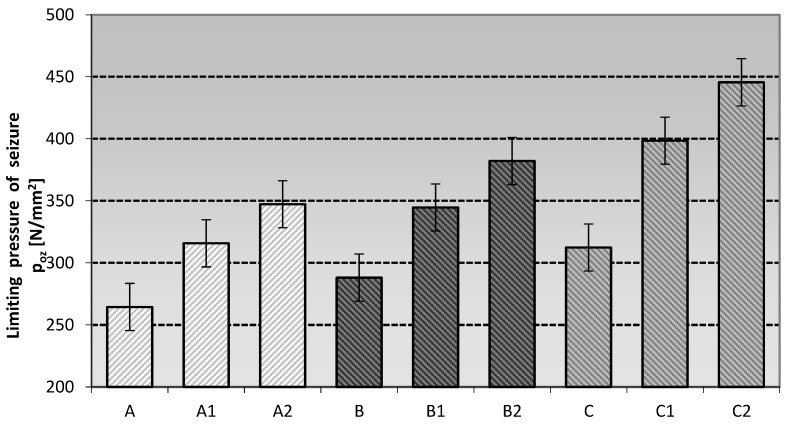
The limiting pressure of seizure of friction nodes with lubricants with different vegetable dispersion phase and modified by silicon nanoadditives. A–C: designation of the tested grease.

The measurement of the anti-seize properties of the lubricants investigated under seizure loads was referred to as the seizure pressure limit (P_oz_). The data obtained for this value are shown in Figure 4.

The anti-seize properties of the tested grease compositions under seizure conditions depend on the type of vegetable-based oil and the amount of additive used, which can significantly or slightly improve/worsen the properties of the resulting lubricants.

The behavior of the modifying nanoadditive is conditioned by many factors, e.g., the method of incorporation into the structure of the grease, the level of its content in relation to the total mass of the produced materials and the type of base oil or thickener used to produce the investigated lubricants. The most advantageous anti-scuffing protection under scuffing conditions is characteristic of soy-based lubricants modified with one of the representatives of layered silicates, namely montmorillonite (C2) and amorphous silica (C1).

Lubricating compositions prepared using linseed and rapeseed oils and modified with nanoadditives did not show such favourable changes in anti-scuffing properties as those prepared using soybean oil. An improvement of the tribological properties is noticeable in relation to the basic compositions without the modifying nanoadditive, but a faster increase was observed for the compositions prepared on the soybean dispersion phase. The lubricants modified with amorphous silica (Aerosil) showed slightly lower anti-scuffing properties than those modified with layered silicates, regardless of the base oil used. The most favourable effect of amorphous silica in improving the anti-scuffing properties compared to the base lubricants was observed for the lubricants based on soybean oil (C1), slightly worse when using linseed oil (B1), and the weakest effect of this modifier was observed for the compositions based on rapeseed oil (A1). On the other hand, the lubricating compositions modified with layered silicate showed the best anti-scuffing properties. The most favourable effect of the layered silicate on the improvement of the anti-seize properties of lubricating greases in comparison with the basic compositions can be observed for greases based on soybean oil (C2), slightly worse for linseed oil (B2), and the weakest effect of this modifier was recorded for the mixtures based on the rapeseed dispersion phase (A2).

The results of the seizure threshold pressure measurements showed that the application of layered silicates in the form of montmorillonite had the best effect on improving the anti-seizure properties of the lubricating compositions used in the experiment. The use of Aerosil-modified silica as a modifier improved the antiscuffing properties relative to the base grease compositions, but had a lower antiscuffing effect than the phyllosilicates. The use of layered silicates in the form of montmorillonite and modified silica in the form of Aerosil in vegetable lubricants, regardless of the base oil used, showed a positive effect of these substances on the resistance of the top layer to scuffing.

The p_oz_ value indicates the pressure in the friction zone during scuffing. Based on the data presented, it can be concluded that the nanoadditives used did not result in the formation of extremely scuffing protective films. The increased poz values of the lubricants containing a soybean dispersion phase and modified with silicon nanoadditives indicate that the nature of the film favours a strong reduction in wear.

**Figure 5:**
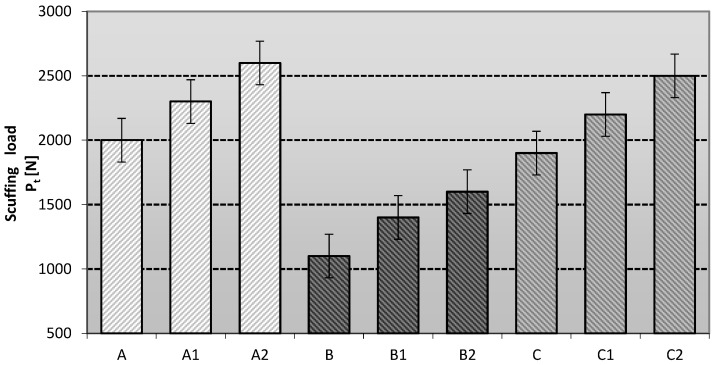
The scuffing load of friction node of lubricants with various vegetable dispersion phase and modified by silicon nanoadditives. A–C: designation of the tested grease.

The anti-scuffing behavior under a linear load, characterized by the scuffing load P_t_, was determined for all the lubricant blends studied. The data are shown in Figure 5.

The scuffing force describes the anti-scuffing behavior of the tested greases under a linearly increasing load. The P_t_ index measures the ability of the lubricating film to transmit stress. Depending on the type of nanoadditive and the vegetable oil base used to prepare the lubricant composition, the value of the scuffing load varied. The highest value of P_t_ was observed for grease A2 based on rapeseed oil modified with montmorillonite and for grease C2 based on soybean oil modified with montmorillonite, while the lowest value of this parameter was shown for lubricants produced on the basis of the linseed dispersion phase. The greases based on rapeseed and soybeans modified with one of the layered silicates, namely montmorillonite (A2 and C2), had the most favourable anti-stick properties under a linearly increasing load. The soy and rapeseed greases modified with amorphous silica (A1 and C1) had slightly weaker properties. The lubricating compositions prepared using linseed oil and modified amorphous silica and montmorillonite did not show such favourable changes in anti-scuffing properties as those prepared using rapeseed or soybean oil. An improvement in the tribological properties was observed with respect to the base compositions without nanoadditive modification, but a faster increase was observed for the compositions prepared with rapeseed and soybean oil. Lubricant compositions modified with amorphous silica (Aerosil) showed slightly lower anti-scuffing properties than those modified with layered silicates, regardless of the base oil used. The most favourable effect of amorphous silica on the improvement of anti-scuffing protection compared to the base lubricants was observed for lubricants based on rapeseed oil (A1), slightly worse when using soybean oil (C1), and the weakest effect of this modifier was observed for compositions based on linseed oil (B1). On the other hand, the grease compositions modified with layered silicate showed the best anti-scuffing properties. The most favourable effect of the layered silicate on the improvement of the anti-scuffing properties compared to the base lubricants was observed for the greases based on soybean oil (C2) and rapeseed oil (A2), while the weakest effect of this modifier was observed for the lubricants based on the linseed dispersion phase.

The type of modifier used influences the change in the anti-seize properties of the tested lubricant compositions. The highest durability of the lubricating film is ensured by the use of layered silicates, regardless of the oil base used—montmorillonite was used as a representative of this type of compound, which allowed us to obtain the lubricant with the highest P_t_ level.

It can be concluded that the efficiency of anti-scuffing is a function of the microstructure of the interface between the additive and the dispersion phase. The individual layered silicate particles in the grease film are more densely interlocked, which increases their ability to interact with each other and thus withstand higher levels of stress.

**Figure 6:**
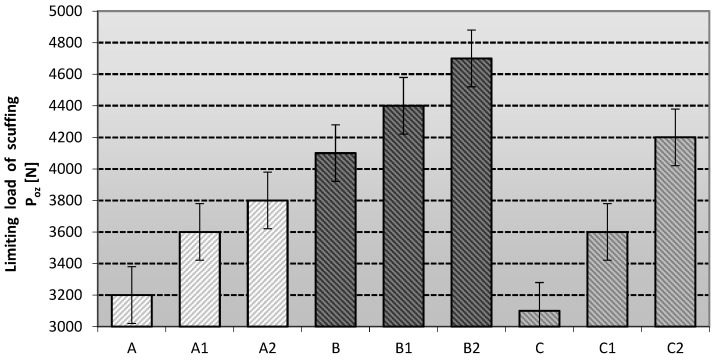
The limiting load of scuffing of friction node lubricated lubricants with various vegetable dispersion phase and modified by silicon nanoadditives. A–C: designation of the tested grease.

The scuffing limit load of the friction point was also determined using the lubricant blends. The data are shown in Figure 6.

The limiting load of scuffing allows the degree of anti-scuffing protection of the lubricants studied to be determined. The best anti-scuffing properties were shown by the linseed-based lubricants modified with one of the phyllosilicates, montmorillonite (B2). Soy-based greases modified with layered silicate (C2) and linseed-based greases modified with amorphous silica (B1) had slightly weaker properties. The lubricating compositions based on rapeseed oil and modified with ceramic nanoadditives did not show such favourable changes in the anti-scuffing properties as the compositions based on the linseed dispersion phase. There was a noticeable improvement in the tribological properties with respect to the basic compositions without the modifying additive, but a faster increase was observed for the compositions prepared in soybean oil. The lubricant compositions modified with amorphous silica (Aerosil) showed slightly lower anti-scuffing properties than those modified with layered silicates, regardless of the base oil used. The most favourable effect of amorphous silica in improving antiscuffing properties compared to the base lubricants was observed for lubricants based on rapeseed and soybean oil (A1 and C1), whereas the weakest effect of this modifier was observed for the compositions prepared in linseed oil. The lubricant compositions modified with layered silicate showed the best anti-scuffing properties of the greases tested. The most favourable effect of the layered silicate on the improvement of the anti-scuffing properties in comparison with the base lubricants can be observed for lubricating greases based on soybean oil (C2), which were slightly less for rapeseed and linseed oil (A2 and B2). The type of modifying additive used influences the change in the anti-scuffing properties of the lubricants studied. The highest durability of the lubricating film was ensured by using layered silicates, regardless of the base oil used—montmorillonite was used as a representative of this type of compound, which allowed us to obtain the grease with the highest value of P_oz_. The P_oz_ values were between 3600–4600 N, which shows that the differences in the lubricant blends had a significant effect only in the case of medium scuffing. The frictional effect leads to an increase in frictional forces in the tribological system, which results in the removal of grease films from the interacting areas of the tribological system. Protection against immobilisation of the friction system can be achieved by using lubricant blends that are compatible with the material of the tribological system.

**Figure 7:**
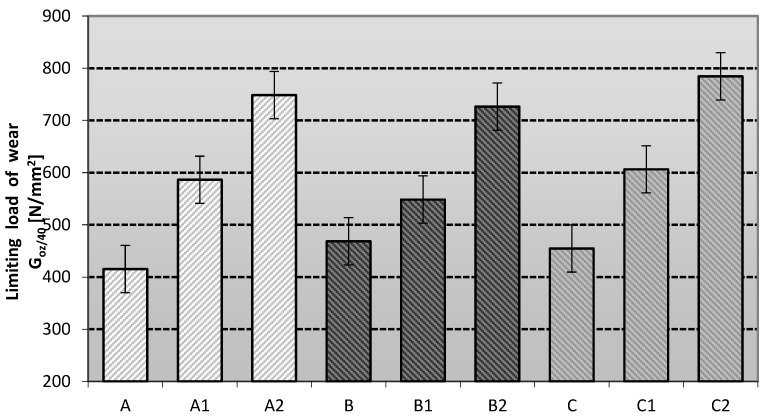
Limiting load of wear of friction node with mixtures lubricated by different vegetable dispersion phases and modified by silicon nanoadditives. A–C: designation of the tested grease.

The antiwear properties of the studied lubricants were evaluated by measuring the limiting wear load G_oz/40_ of the friction node with mixtures lubricated with different vegetable dispersion phases and modified with silicon nanoadditives, and the obtained data were analyzed. The results obtained are shown in Figure 7.

The limiting wear load makes it possible to determine the level of anti-wear properties of the lubricants under testing. The level of stabilization of the film is defined by the index of the limiting load of wear G_oz/40_. The higher the value, the better the stability of the lubricant and its anti-wear properties. The highest value of this parameter was observed for the linseed-based lubricant (B), slightly lower for the soy-based lubricant (C), and the lowest value of this parameter represents the rapeseed-based lubricant (A). Soy-based lubricants modified with one of the phyllosilicates, montmorillonite (C2), had the best anti-wear properties. The rapeseed-based lubricants modified with montmorillonite (A2) were characterized by slightly weaker properties, and the weakest effect of the layered silicate was observed for the composition prepared in linseed oil (B2). An improvement of the tribological properties was noticeable with respect to the basic compositions without the modifying additive, but a faster increase was observed for the compositions prepared in soybean and rapeseed oil. The lubricating compositions modified with amorphous silica (Aerosil) showed weaker antiwear properties than the tested lubricants modified with layered silicates, regardless of the base oil used. The most favourable effect of amorphous silica in improving the antiwear properties in comparison with the base compositions was observed for the lubricants based on soybean oil (C1), slightly worse in the case of using rapeseed oil (A1), and the weakest effect of this modifier was obtained for lubricants based on the linseed dispersion phase (B1). The comparison with the lubricants based on the vegetable dispersion phase modified with nanoadditives showed an improvement in the general antiwear properties, which was not as clear for the lubricants based on synthetic or mineral dispersion phase [29].

The type of modifying additive used influences the modification of the antiwear properties of the lubricant formulations studied. The highest antiwear performance was obtained by using layered silicates, regardless of the base oil used. The specific requirements for greases in the agro-food sector are defined according to the individual needs of the machine and equipment manufacturers. According to customer studies, lubricants are categorised as follows: those with G_oz/40_ > 600 N/mm^2^ have excellent anti-wear properties, those between 400 and 600 N/mm^2^ are considered effective and those with G_oz/40_ less than 400 N/mm^2^ are considered inadequate. The level of antiwear properties obtained demonstrated that all the lubricant compositions tested were effective under a constant tribological load.

In the work of Adhvaryu et al. [50], results were obtained showing that the use of montmorillonite in lubricants reduced the coefficient of friction and improved the viscosity–temperature properties. In the work of Hussein et al. [51], the authors demonstrated that the use of nanosized silica significantly reduced the coefficient of friction and improved the rheological properties, including viscosity versus temperature and shear rate curves, flow curves and hysteresis areas of the lubricants tested. In the work of Nosov and Kamenskikh [106], the authors demonstrated an improvement in the tribological and rheological properties of vegetable greases under the influence of the addition of nanometric silica. In the work of Uppar et al. [107], the authors demonstrated that the introduction of nano-additives into the structure of bio-lubricants improves the rheological properties and significantly reduces wear. In the work of Shahabuddin et al. [108], the authors obtained a significant reduction in the viscosity and coefficient of friction of vegetable-based plastic greases after removing the addition of nanometric amorphous silica. In the work of Jabal et al. [109], the authors demonstrated that the use of nanoadditives reduces the coefficient of friction and the viscosity–temperature properties of vegetable-based plastic greases. In work of Liu H. et al. [110], the authors demonstrated that the introduction of nanoadditives into the structure of greases reduces the coefficient of friction and improves the rheological properties. In the work of Quinchia et al. [111], the authors demonstrated that nanoadditives introduced into the structure of vegetable greases reduced the coefficient of friction and improved the lubricating properties of the tested lubricating compositions. In Bao et al. [112], the authors investigated the effect of SiO_2_ nanoparticles on the tribological properties of lubricants. The authors obtained a significant improvement in the anti-wear properties and a reduction in the coefficient of friction. 

### 3.2. Results of Classical Rheology Tests 

**Figure 8:**
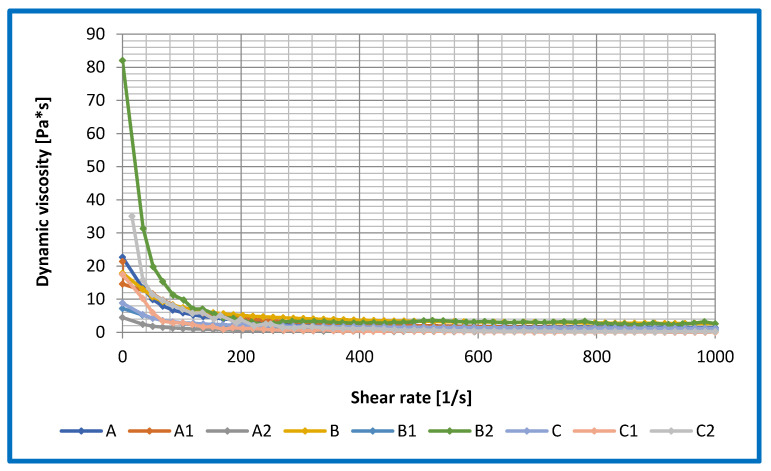
The dependence of dynamic viscosity on shear rate for basic lubricants with different vegetable oil bases and compositions modified by silicon nanoadditives.

The relationship between dynamic viscosity and shear rate was determined for the base lubricants and lubricants modified with ceramic nanoadditives. The results obtained are shown in Figure 8.

The tests were carried out to determine the appropriate shear rate for the lubricant compositions tested. As a result of the research conducted, it was observed that the shear rate in the range of 0–1000 [1/s] best illustrates the differences between the individual lubricant compositions. For the compositions based on vegetable oils, a positive effect of the applied nanoadditive in the form of amorphous silica and montmorillonite on changes in the dynamic viscosity at high shear rates was observed; the viscosity value decreased in comparison to the base composition without additives. The introduction of layered silicate in the form of montmorillonite into the structure of grease based on vegetable oils showed a negative effect of the used additive on viscosity changes at a low shear rate, which was caused by insufficient distribution of the used additive in the structure of the tested lubricant. Too short of a homogenization time could cause significant changes in the dynamic viscosity at low shear rates for the tested greases.

At lower shear rates, the dynamic viscosity of the rapeseed dispersion phase-based lubricant was lower than the dynamic viscosity of the silica- or montmorillonite-modified lubricants. The use of the silica nanoadditive in the rapeseed-based lubricant had a positive effect on the change in viscosity values at a low shear rate. No major changes in the dynamic viscosity at high shear rates were observed for the rapeseed oil-based greases. The lower viscosity of the obtained lubricating composition based on rapeseed oil may lead to the possibility of using such a lubricant in many branches of industry, where until now there have been major problems in lubricating the components of equipment and machines with greases of much higher viscosity than the investigated mixtures.

For the compositions based on linseed oil and modified with amorphous silica, the dynamic viscosity at the lower shear rate value was lower than the dynamic viscosity of the base composition without a modifier. However, at higher shear rates, the dynamic viscosity for linseed oil-based formulations did not show significant changes in dynamic viscosity. For the linseed oil-based grease modified with montmorillonite, the dynamic viscosity at lower shear rates was almost four times that of the base composition without a modifier. This situation is not favourable in many industries in central lubrication systems because the grease has to overcome the flow resistance and can cause significant energy losses. At a high shear rate, the dynamic viscosity of the montmorillonite-modified grease did not change significantly.

On the other hand, for lubricants based on the soybean dispersion phase, the dynamic viscosity at a low shear rate was higher than for the compositions based on linseed and rapeseed oil, which may be due to the chemical structure of the base oil used. The use of amorphous silica as an additive reduced the dynamic viscosity in the low shear rate range compared to the results obtained for the base composition. At higher shear rates, no significant changes in dynamic viscosity were recorded for this composition. At lower shear rates, the dynamic viscosity of the montmorillonite-modified soybean oil-based composition was twice that of the base composition without additive. At higher shear rates, the dynamic viscosity of the montmorillonite-modified blend was equal to that of the base blend, and no significant change was observed. 

The evaluation of the changes in dynamic viscosity for the lubricants studied showed that the use of vegetable base oils as the dispersing phase of the lubricants resulted in significant differences in viscosity at low shear rates. The level of dynamic viscosity of the investigated lubricants was varied according to the type of dispersant, its chemical structure and the method and time of homogenisation. The lowest values of dynamic viscosity in the regime of low shear rates were characterised by the mixture prepared on the basis of the rape seed dispersion phase and modified by layered silicate.

**Figure 9:**
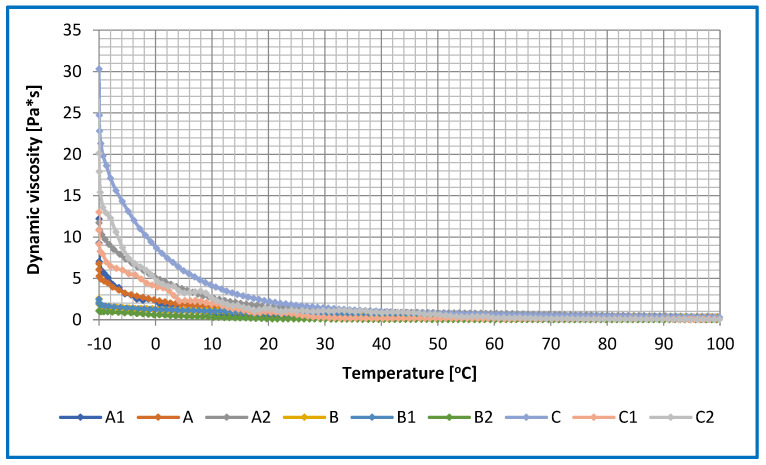
The dependence of dynamic viscosity on temperature for basic lubricants with different vegetable oil bases and compositions modified by silicon nanoadditives.

The dependence of dynamic viscosity on temperature was determined for basic lubricants and lubricants modified with ceramic nanoadditives. The viscosity curve was determined in the temperature range −10–100 °C and at a shear rate equal to 1000 s^−1^. The results obtained are shown in Figure 9.

At temperatures between 30 °C and 100 °C, no significant changes in dynamic viscosity were observed for any of the lubricants tested. The higher viscosity at lower temperatures was observed for the soybean oil-based composition. The introduction of amorphous silica and montmorillonite into the soybean oil-based grease caused a decrease in the dynamic viscosity at lower temperatures. A positive effect of the used additive on the rheological properties of vegetable greases depends on the physico-chemical properties of soybean oil and its chemical structure. 

For lubricants based on the linseed dispersion phase, the dependence of dynamic viscosity in a wide range of temperatures was determined. By modifying the structure of montmorillonite, it is possible to obtain a composition with a minimum value of dynamic viscosity at low temperatures, which is very important in many industrial sectors. The use of amorphous silica as a modifier in the linseed dispersion phase allows for good rheological properties to be maintained at low temperatures.

The relationship between the dynamic viscosity and thermal expansion for rapeseed oil-based lubricants is similar to that for linseed oil-based greases. The addition of amorphous silica to the greases resulted in an increase in the dynamic viscosity at low temperatures, which is not a positive phenomenon. The application of montmorillonite, on the other hand, caused a significant decrease in the dynamic viscosity at low temperatures, which is favourable in many branches of industry, especially when starting up machines and equipment in many technological processes. This shows that the use of vegetable oils as a disperse phase of lubricants strengthens the physical structure of such lubricants and reduces the probability of the occurrence of negative effects, which can cause changes in the quality of the applied mixture. It is of great importance when selecting the design criteria of equipment and machinery in many industries, for example when developing centralized lubrication systems. All the lubricants presented in this paper are non-Newtonian media with dynamic viscosities that decrease with temperature and shear rate, i.e., they are thinned by shearing. The rheological properties of lubricant blends are influenced by the nature of the disperse phase, the dispersed nature of the phase, the lubricant manufacturing technology and the operating environments in which the lubricant is used. The relationship between the dispersed phase and the particles of the dispersed phase will increase as the amount and type of base oil increases until a critical optimum is reached. As this interaction and the nature of the dispersed phase change, the level of dynamic viscosity depends on the temperature, shear rate and yield stress of the lubricant. 

The conditions under which the lubricant is manufactured have a major influence on the durability of its structure and the formation of tangential stress limits. In order to stabilize this structure and make it more coherent and resistant to external influences, oil bases are used, which influence the value of the free potential energy between the liquid and solid phases in the desired range.

**Figure 10:**
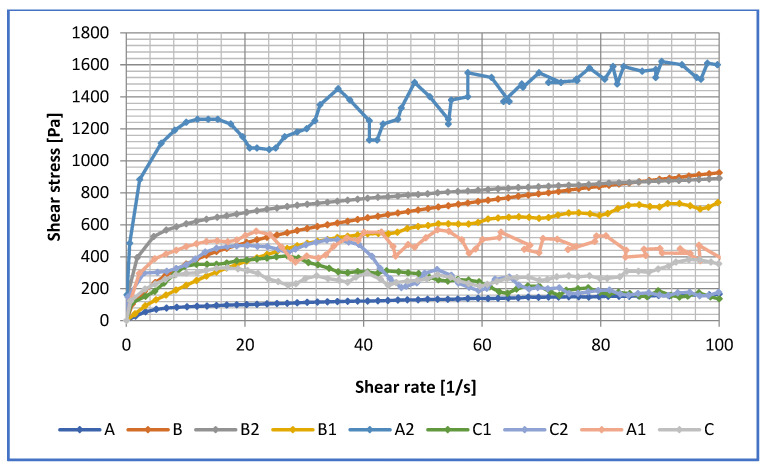
The dependence of shear stress on shear rate (flow curves) for basic lubricants with different vegetable oil bases and compositions modified with silicon nanoadditives.

**Figure 11:**
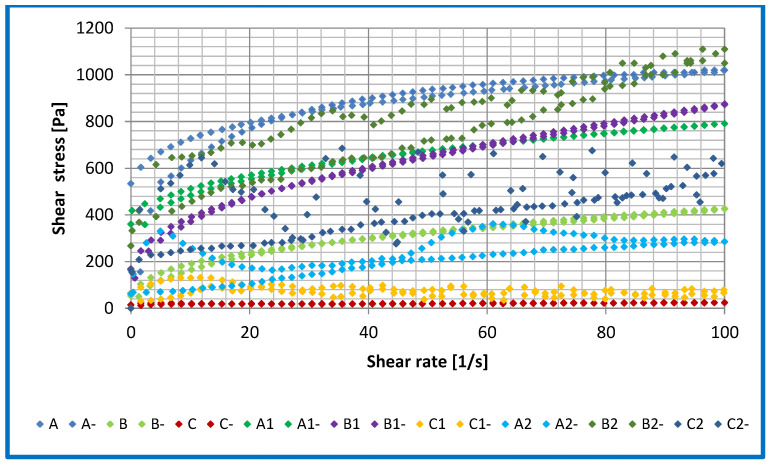
Shear stress as a function of increased and decreased shear rate (flow curves) for basic lubricants with different vegetable oil bases and compositions modified with silicon nanoadditives.

The data presented in Table 5a–d and Figure 10 and Figure 11 show the experimentally determined flow curves of the lubricant compositions tested and the parameters and correlation coefficients calculated using the four rheological models—Casson, Bingham, Herschel–Bulkley and Tscheuschner. The area of loop hysteresis, i.e., between the flow curve with increasing shear rates and the flow curve with decreasing shear rates, was calculated using the integral method. The values obtained were used to determine the thixotropic properties of the lubricants tested and are presented in Table 6.

For greases A, B and C, which were not modified with the additives used (amorphous silica and montmorillonite) (Figure 9 and Figure 10 and Table 4), the best mapping of the flow curves determined experimentally was obtained using the Herschel–Bulkley, Casson and Tscheuschner models, which were characterized by high correlation coefficients (Table 4), compared to the Bingham model, which did not describe the flow curves of these greases very well. No significant differences were found between the yield point values for these compositions obtained from the four rheological models. The tests of structural viscosity carried out using the four applied rheological models for greases based on rapeseed, linseed and sunflower oils, which were not modified with additives, did not show any significant differences in the level of this indicator.

For lubricant A1 based on rapeseed oil modified with amorphous silica, the R^2^ coefficient and the structural viscosity decreased, while the value of the yield point for this composition increased approximately six times. The modification of the rapeseed-based grease with montmorillonite (A2) caused a significant decrease in the R^2^ coefficient and the structural viscosity (four times), while the value of the yield point increased approximately 17 times. These results indicate changes in the structure of the lubricants tested. The reason for such changes may be the way in which the modifying additive was incorporated into the structure of the lubricant. The synergistic interaction of the base oil with the additive causes a significant change in the rheological properties, characterized by a change in the value of the structural viscosity and the yield point of the rapeseed oil-based lubricants.

For grease B1 produced using linseed oil modified with amorphous silica, a decrease in the R^2^ coefficient and a significant decrease in the yield point (approximately 38%) and structural viscosity (more than two times) were observed. The introduction of montmorillonite into the composition of the structural grease caused an increase in the yield point (approximately 38%) and a more than fourfold increase in the value of the structural viscosity. The chemical structure of used additives in comparison with the chemical structure of linseed-based oils could cause significant changes in the calculated rheological parameters.

The compositions created by introducing silica into the structure lubricants (C1) caused an increase in the R^2^ coefficient, yield point (approximately 39%) and structural viscosity (approximately two times). The modification of the lubricant structure by montmorillonite (C2) caused a decrease in the R^2^ coefficient and yield point (approximately 14%) and an increase in the value of the structural viscosity of approximately four times. The structure created by the base oil, thickener and additive and their properties caused a change in the rheological parameters, resulting in a change in their useful properties and the structure of the soybean oil-based grease.

The area of the hysteresis loop is a measure of the thixotropy of the lubricants tested. The mechanism of thixotropy is related to the phenomenon of shear thinning. The phenomenon of thixotropy can be explained by the aggregation of thixotropic fluid particles. When the grease is subjected to shearing, the structure created can be partially or completely disintegrated. After some time, an equilibrium is reached which defines a new state of the intermolecular bonding network in lubricants. All the vegetable lubricants tested, modified with amorphous silica and montmorillonite to different degrees, showed the phenomenon of thixotropy, leading to an overlapping of the flow curves obtained with increasing and decreasing shear rates. The values of the hysteresis loop surface areas of the lubricants are shown in Table 4. The modification of the vegetable grease with montmorillonite caused a significant increase in the size of the hysteresis area (from 2 to 15 times, depending on the oil base), which is not a favourable phenomenon because the reconstruction of the damaged structure will require the application of large forces, requiring a significant energy content. On the other hand, the use of amorphous silica as a modifier resulted in a reduction in the hysteresis surface by approximately 40% for rapeseed oil-based greases and an increase in the hysteresis surface by 50–175% for linseed and soybean oil-based greases. The larger the hysteresis area, the weaker the thixotropic properties and the lower the ability to restore the damaged structure of the grease composition.

The highest value of the thixotropy hysteresis surface was observed in the vegetable lubricant based on soybean oil and modified with montmorillonite, while the lowest value was observed in the greases based on rapeseed and linseed oil and in the lubricants modified with amorphous silica. The most effective action of montmorillonite was observed for the composition based on the rapeseed dispersion phase, and the best action of amorphous silica was obtained for the linseed oil lubricant phase, which restored the damaged structure the fastest.

Lazaro and Aranda [45] carried out rheological tests on vegetable soap greases and obtained greases with very good structural recovery results after tribological tests. Ta-ha-Tijerina et al. [46] obtained satisfactory results for SiO_2_-modified greases, characterised by a significant reduction in the coefficient of friction and an improvement in the rheological properties. The work of Martín-Alfonso et al. [48] investigated the effects of montomorillonite on the rheological properties of vegetable lubricants. The resulting lubricants were characterized by a significant improvement in rheological properties, such as structural recovery, as well as a reduction in the coefficient of friction. And in paper [49], the authors used montmorillonite to modify vegetable lubricants. According to the results obtained, montmorillonite improved the tribological, rheological and microstructural properties of the lubricants obtained. Zakani et.al [113] studied the rheological properties of a grease modified with amorphous silica. The authors obtained a significant improvement in the viscosity and viscoelastic properties of the lubricant with the addition of nano-SiO_2_. In another paper [114], the authors showed that the addition of amorphous silica improves the viscoelastic and viscosity–temperature properties of vegetable greases. In the work of Martin-Alfonso et al. [115], the effect of amorphous silica on the rheological properties of lubricants may be overestimated. The authors showed that the addition of silica improved the tribological and rheological properties, including the anti-wear, viscosity–temperature and viscoelastic properties of vegetable-based lubricants. A paper by Gómez-Merino et al. [116] investigated the effects of nanosized silica on the rheological properties of vegetable-based lubricants. Improvements in viscosity and tribological properties as well as microstructural properties at high loads were obtained. Kumar and Garg in their work [117] investigated the tribological properties of vegetable-based lubricants. The authors showed that the introduction of nano-additives into plant-based lubricants improved rheological properties such as dynamic viscosity at low shear rates and temperatures and reduced the coefficient of friction.

### 3.3. Results of Optical Rheology Tests

**Figure 12:**
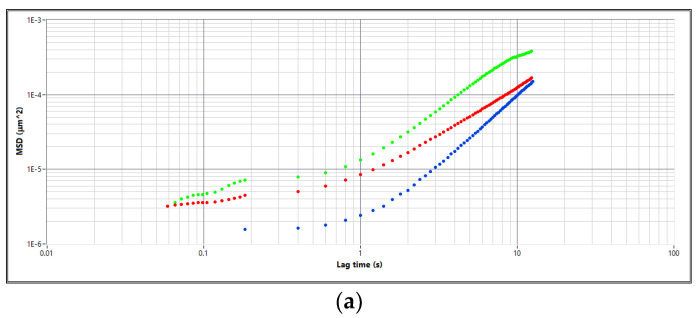

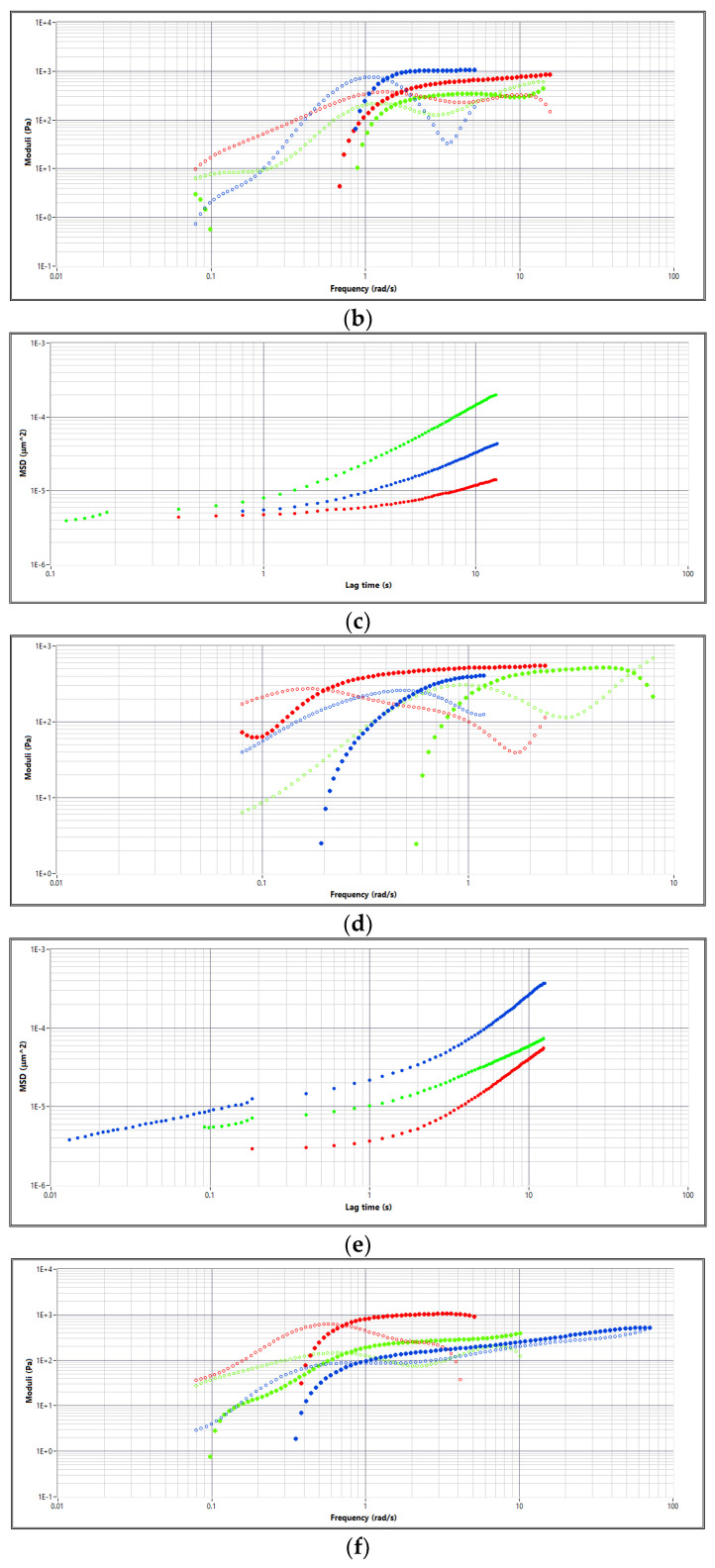
(**a**) The effects of silicon additives on the rheological properties of vegetable lubricants: relationship between mean square displacement curve and lag time (blue—rapeseed-based lubricant, red—rapeseed-based lubricant modified with silica additive, green—rapeseed-based lubricant modified with montmorillonite). (**b**) Effect of silicon additives on the rheological properties of vegetable lubricants: relationship between the elastic curve (G′) and the viscous curve (G″) as a function of frequency (blue—rapeseed-based lubricant, red—rapeseed-based lubricant modified with silica, green—rapeseed-based lubricant modified with montmorillonite). (**c**) The effect of silicon additives on the rheological properties of vegetable lubricants: relationship between mean square displacement curve and lag time (blue—linseed-based lubricant, red—linseed-based lubricant modified with silica, green—linseed-based lubricant modified with montmorillonite). (**d**) Effect of silicon additives on the rheological properties of vegetable lubricants: relationship between the elastic curve (G′) and the viscous curve (G″) as a function of frequency (blue—linseed-based lubricant, red—linseed-based lubricant modified with silica, green—linseed-based lubricant modified with montmorillonite). (**e**) The effect of silicon additives on the rheological properties of vegetable lubricants: the relationship between the mean square displacement curve and the deceleration time (blue—soybean oil-based lubricant, red—soybean oil-based lubricant modified with silica additive, green—montmorillonite-modified soybean oil-based lubricant). (**f**) The effect of silicon additives on the rheological properties of vegetable lubricants: the relationship between the modulus of elasticity (G′) and the viscosity coefficient (G″) as a function of frequency (blue—soy-based lubricant, red—soy-based lubricant modified with silica, green—soy-based lubricant modified with montmorillonite).

The rheological parameters of the investigated plant lubricants were determined and evaluated before and after the introduction of silicon-modified additives into their structure, and the effect of a silicon additive on the evolution of the rheological properties of the investigated lubricants was evaluated. The experimental data are shown in Figure 12a–f.

A particle size study of Aerosil 300 amorphous silica was carried out using the DWS technique, and the silica particle size was in the range of 28–32 nm. A particle size study of the layered silicate montmorillonite was also carried out, and the particle size was in the range of 120–130 nm. The dependence of the mean square displacement (MSD) function on the lag time for the tested greases is characterised by the presence of three phases, depending on the type of base oil and the type of thickeners in vegetable greases. At very low lag times of the MSD correlation function, the increase in the discussed function depends on the type of thickeners used in the tested greases. At greater lag times, higher values of the MSD function were observed, which proves the diffusive nature of the tested greases with a slope of the correlation function close to unity. In the range of lag times from 2 × 10^−1^ to 4 × 10^−1^ s, the tested function is characterised by the so-called plateau. No significant changes in the value of the MSD curve were observed during this period. When analyzing the shape and values of the correlation function with different types of thickeners, it should be noted that the value of the function increases with increasing the lag times, which proves that the elastic properties of the tested samples change towards higher values. However, in the range of 6.0 × 10^−1^ to 1.2 × 10^1^ s, the value of the MSD curve increases, which proves the subdiffusion properties of the tested greases prepared using different types of thickeners. In the case of the grease prepared using linseed oil, the values of the MSD function changed significantly at higher lag times. Comparing the test results with the greases prepared using linseed oil, it can be observed that the MSD function takes higher values at higher lag times. On the other hand, the plateau assumes values similar to those of the rapeseed oil base, indicating a change in the elastic properties as well as a change in the viscosity of the tested grease. In the range of low lag times (1.0 × 10^−1^ ÷ 8.0 × 10^−1^ s), the MSD function reaches values similar to the values of this parameter for grease with rapeseed oil; therefore, the viscosity of the tested grease does not change significantly.

In the case of the greases prepared using soybean oil, lower values of the mean square dispersion curves were observed in connection with low lag times (1.0 × 10^−2^ ÷ 1.0 × 10^1^ s) in comparison with the data obtained for the greases prepared using the linseed and rapeseed dispersion phase, indicating a modification in the diffusion characteristics of the studied greases. The plateau value of this grease is higher than that of the greases based on rapeseed and linseed oil and lower for the montmorillonite-modified grease than for the base grease based on soybean oil. These plateau values increase the flexibility and viscosity of the lubricants evaluated. However, in the range of higher lag times (8.0 × 10^−1^ ÷ 1.2 × 10^1^ s), the MSD function takes higher values than for the greases with linseed and rapeseed oil, which demonstrates the much stronger properties of the tested greases. The tests carried out on greases modified with amorphous silica and montmorillonite showed that in the range of low lag times (1.0 × 10^−2^ ÷ 1.0 × 10^1^ s), the MSD function assumes lower values compared to the results of the tests carried out on base greases on soybean-based oil, which is proved by weaker diffusion properties of the tested lubricant and, therefore, a tendency to increase the viscosity. When analyzing the middle range of the lag time, i.e., the plateau of the correlation function, a decrease in the value of the correlation function was observed in relation to those for base greases with linseed and rapeseed oil, which led to an increase in the strength of the elastic properties and a change in the viscosity of the lubricant studied. At higher lag times (8.0 × 10^−1^ ÷ 1.2 × 10^1^ s), a decrease in the values of the MSD function was observed for greases modified with amorphous silica and montmorillonite in comparison with greases without additives, which means that the modified greases have a high stability to changes in viscosity. The results obtained allow us to state that the greases modified by ceramic additives are characterized by stranger viscous properties than greases without additives, which could be caused by the change in the microstructure of the sample in question as a result of the processes of degradation of the oil base and the oxidation products formed.

The rheological tests carried out on vegetable greases based on rapeseed oil show that the modulus of elasticity dominates the modulus of viscosity over a wide frequency range (8.0 × 10^−1^ ÷ 1.20 rad/s), which is a characteristic behavior for a solid. The tests carried out on the tested grease based on rapeseed oil and modified with amorphous silica showed that a decrease in the value of the storage modulus of 19% was observed in comparison with the results of the tests carried out on the basic grease based on rapeseed oil. 

For the greases modified with montmorillonite, significant changes in the value of the modulus were observed in the low frequency range (below 6.70 × 10^0^ rad/s), which is characteristic of the diffusion behavior in the dispersed system under resistivity. Analyzing the loss modulus G″, it should be noted that significant differences in the value and shape of this modulus were observed for vegetable greases with rapeseed oil and greases modified by ceramic additives in the frequency range above 2.15 × 10^1^ rad/s. It was observed that greases modified with silica and montmorillonite were characterized by much lower values than the basic rapeseed grease. Changes in the viscous modulus (G″) were observed in the low frequency range (below 1.83 × 10^1^ rad/s). 

For the grease prepared using linseed oil, the value of G″ increased significantly up to the frequency of 1.26 rad/s. In the range of 8.0 × 10^−2^ ÷ 5.2 × 10^−1^ rad/s, the G″ modulus dominated the G′ modulus, showing the predominance of viscous properties. At higher frequency values, the G′ moduli were characterised by higher values. However, for lubricating grease based on linseed oil and modified with amorphous silica, the structure was changed because the values of G″ modulus were much higher than the values of G′ modulus up to 2 × 10^−2^ rad/s. Above this frequency, the G′ modulus had much larger values than the G″ modulus. This result indicates strong viscous properties for the greases tested. In the case of montmorillonite-modified greases, the loss modulus (G″) in the frequency range 9.3 × 10^−2^ ÷ 1.20 × 10^1^ rad/s and above 6.40 × 10^1^ rad/s was observed for higher values of the discussed parameter in relation to the values of the G′ modules. This behavior of the tested grease indicates a stable viscosity in the tested frequency range. In the frequency range between 1.20 × 10^1^ and 6.40 × 10^1^ rad/s, a characteristic minimum of this function was observed at 4.40 × 10^1^ rad/s, which may indicate changes in the structure of the tested sample.

For grease prepared using soybean oil, the rheological properties were determined, and it was observed that in a wide frequency range (9.12 × 10^−1^ ÷ 73.4 × 10^1^ rad/s), they were characterised by higher values of the G″ modulus than the value of the G″ modulus. At lower values of frequency (below 0.85 rad/s), the G″ modules dominated. This situation shows that the elastic forces of the lubricant increased with increasing frequency, which in turn altered its stability. In the case of grease with soybean oil and amorphous silica, the loss modulus had higher values than the values of the storage modulus, above 7.23 × 10^−1^ rad/s. In the range of 7.03 × 10^−2^ ÷ 7.23 × 10^−1^ rad/s, the storage modulus dominates, which indicates the stable and strange viscosity properties of the tested lubricant composition.

For the composition prepared using soybean oil and modified by montmorillonite, there were lower values of the modulus. It was observed that in the range of lower frequencies (7.94 × 10^−2^ ÷ 7.53 × 10^−1^ rad/s), the lower values of the modulus characterized the viscous properties. When increasing the frequency above 7.5 × 10^−1^ rad/s, an increase in the value of this parameter was observed. On the other hand, it was observed that the G′ function initially increased at lower frequency values and then decreased as the frequency value increased. The appearance of the minimum loss modulus in the case of the tested lubricant modified with montmorillonite is characteristic, as this phenomenon has been observed for vegetable greases modified with montmorillonite. The minimum value of the modulus G′ can be influenced by changes in the structure of the tested grease, which occur in the tested sample under the influence of the applied additive.

### 3.4. Spectral Test Results

**Figure 13:**
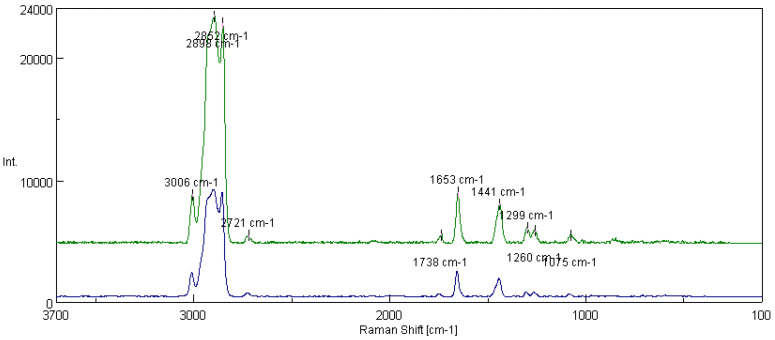
Dependence of integral intensity bands on wave number for a rapeseed dispersion phase lubricant with soap thickener (blue) and modified with Aerosil (green).

**Figure 14:**
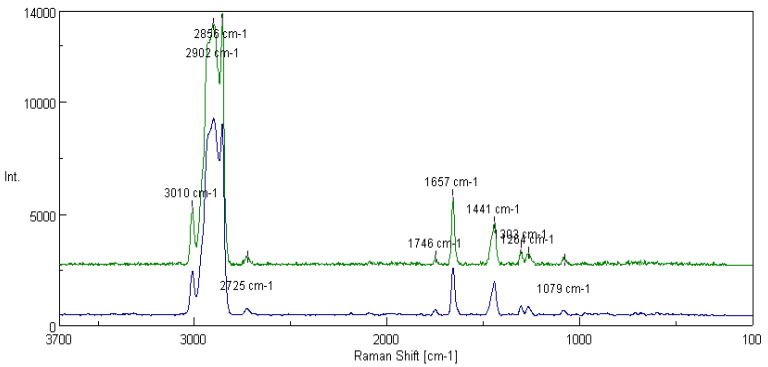
Dependence of integral intensity bands on wavenumber for rapeseed dispersion phase lubricant with soap thickener (blue) and modified with montmorillonite (green).

**Figure 15:**
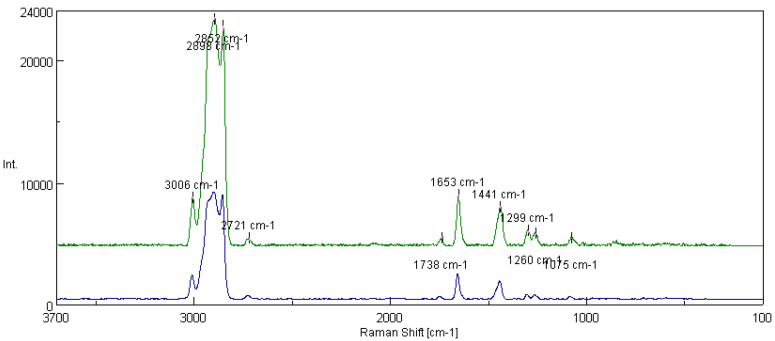
Dependence of integral intensity bands on wave number for a linseed oil-based lubricant composition with soap thickener (blue) and modified Aerosil (green).

**Figure 16:**
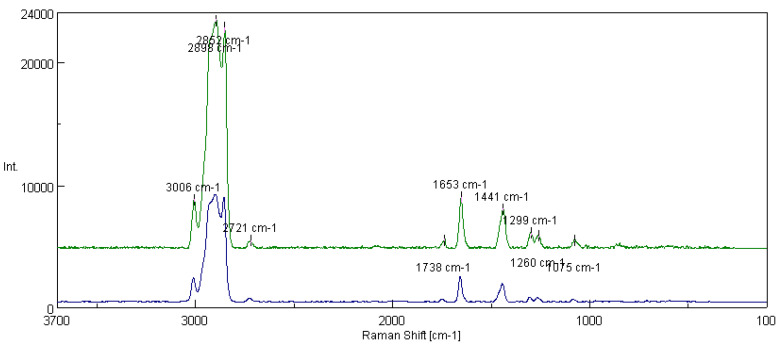
Dependence of integral intensity bands on wavenumber for lubricant compositions prepared from the linseed dispersion phase with soap thickener (blue) and modified with montmorillonite (green).

**Figure 17:**
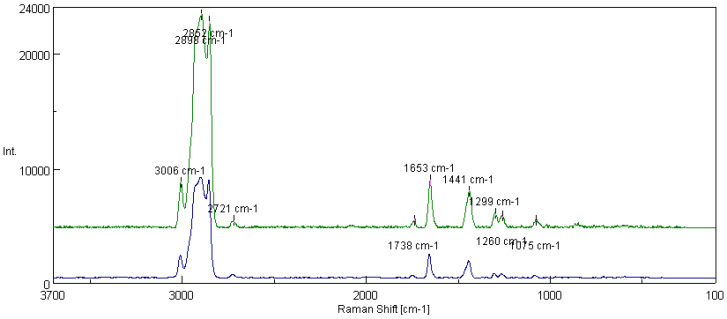
Dependence of integral intensity bands on wave number for soybean oil-based lubricant composition with soap thickener (blue) and modified Aerosil (green).

**Figure 18:**
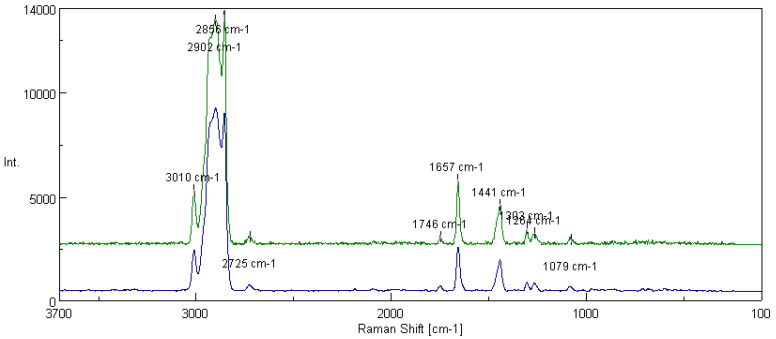
Dependence of integral intensity bands on wave number for soy-based lubricant with soap thickener (blue) and modified with montmorillonite (green).

The chemical structure of the prepared lubricant compositions was determined using a Raman spectrometer. The compositions prepared from the rapeseed, linseed and soybean dispersion phases thickened with lithium stearate and compositions modified with Aerosil 300 amorphous silica and montmorillonite were tested. The results of the spectroscopic tests for the lubricant compositions are shown in Figure 13, Figure 14, Figure 15, Figure 16, Figure 17 and Figure 18.

One of the methods used to obtain information on changes occurring at the molecular level in vegetable-based oils, which are the main component of vegetable lubricants, is to monitor changes based on the characteristic bands present in fatty acids of vegetable oils, such as:

(1) Bands excited by stretching vibrations of the C=C groups, shear vibrations of the CH_2_ group at 1657 and 1441 cm^−1^, respectively,
(2) bands of stretching vibrations =C-H and C-H of CH_3_ and CH_2_ groups appearing at 3003 and 2859 cm^−1^.

The bands excited by stretching vibrations of unsaturated C=C groups at 1657 cm^−1^ and saturated CH_2_ groups at 1441 cm^−1^ were selected for the analysis of changes in the structure of the tested lubricants occurring under the influence of modified additives (amorphous silica, montmorillonite) because these bands are clearly separated, and their boundaries are clearly marked. The intensity of the peak integral was determined as the value of the integral corresponding to the area under the peak. The ratio of the number of C-C single bonds to C=C double bonds present in the fatty acids of vegetable oils was determined, and thus the effect of the additives on the changes in the structure of the vegetable oil was estimated by evaluating its degree of unsaturation. The integration intensity of the I_1441_/I_1657_ bands resulting from the vibrations of the characteristic unsaturation bonds present in vegetable oil was calculated, indicating the degree of its unsaturation.

The integral intensity of the bands (Iint) was calculated from Formula (7):

(7) $$I_{int}=P_{1441}/P_{1657}$$

where:

P—surface area under the peak at 1657 cm^−1^, mm^2^

P—surface area under the peak at 1441 cm^−1^, mm^2^

In the spectrum of the tested lubricants, an increase in the intensity of the bands characteristic of functional groups of polyunsaturated fatty acids was observed in the following ranges: 2985–3036 cm^−1^, 2865–2984 cm^−1^ and 2803–2865 cm^−1^, which proves the existence of changes in the structure of the lubricants as a result of the action of the additives used. It has been assumed that the measure of changes in the structure of the base oil is the area under the peak.

Figure 13 shows the spectra of fats obtained from the rapeseed dispersion phase and modified structure of amorphous silica. In addition to the peaks characteristic of functional groups of polyunsaturated fatty acids, the presence of peaks characterizing the presence of silicon derived from the modifying additive (1075 cm^−1^) and the Si-O bond (1299 cm^−1^) were observed. Based on the tested lubricant compositions, the integral intensity of the characteristic bands of the vegetable base oil (I_1441_/I_1653_) was calculated. The analysis of the obtained data shows that with the introduction of the additive into the vegetable lubricant based on rapeseed oil, the intensity of the characteristic bands for the oil base I_1441_/I_1653_ increased from 0.530 for the lubricating composition not modified with silica nanoadditive to 0.672 for the composition modified with flame amorphous silica Aerosil 300.

Figure 14 shows the spectra of the basic vegetable grease formed on the rapeseed-based oil and thickened with lithium stearate and the rapeseed grease modified with one of the layer silicates, montmorillonite. For the lubricating composition modified with the addition of montmorillonite, peaks derived from Si-O bonds (1264 cm^−1^ and 1079 cm^−1^) were noted in the spectra. For the tested lubricant compositions, the integral intensity of the characteristic bands of the vegetable oil base (I_1441_/I_1657_) was calculated. The analysis of the obtained data shows that with the introduction of montmorillonite into the lubricant structure, the value of the intensity characteristic bands increased from 0.530 for the lubricating composition not modified with the addition of montmorillonite to 0.842 for the composition modified with layered silicate.

Figure 15 shows the spectra of greases prepared from the linseed dispersion phase without modifiers and with the modified structure of Aerosil. In this case, peaks derived from the characteristic Si-O bonds (1075 cm^−1^ and 1299 cm^−1^) were observed, indicating that the additive modified the structure of the lubricant. For the tested lubricant compositions, the integral intensity of the characteristic bands of the vegetable oil base (I_1441_/I_1653_) was calculated. An analysis of the data showed that for the grease modified with the additive, the intensity of the bands increased from 0.582 for the base composition without the additive to 0.756 for the grease modified with amorphous silica.

Figure 16 shows the spectra of fats prepared from the linseed dispersion phase without modifier and with a modifying additive in the form of montmorillonite. In addition to the characteristic bands of the vegetable oil base (3006 cm^−1^, 2898 cm^−1^, 1441 cm^−1^ and 1653 cm^−1^), the spectra were characterized by peaks corresponding to the presence of compounds containing silicon (1299 cm^−1^, 1260 cm^−1^ and 1075 cm^−1^, characteristic of Si-O bonds). For the tested lubricant compositions, the integral intensity of the bands characteristic of the vegetable oil base (I_1441_/I_1653_) was calculated. The analysis of the obtained data shows that the grease modified with montmorillonite was characterized by a coefficient equal to 0.889, which is much higher than the coefficient characterizing the integral intensity of the characteristic bands, which amounted to 0.582.

Figure 17 shows the spectra of lubricating compositions based on soybean oil without modifying additive and modified with amorphous-fumed silica. For the grease modified with the silica additive, the presence of peaks associated with silica structures derived from the additive (1299 cm^−1^, 1260 cm^−1^ and 1075 cm^−1^) was noted in the spectrum. For the tested lubricant compositions, the integral intensity of the characteristic bands of the vegetable-based oil (I_1441_/I_1653_) was determined. The analysis of the obtained data shows that the grease unmodified with the additive is characterized by the coefficient of the integral intensity of the bands at a level of 0.613. The introduction of amorphous silica into the structure of the lubricant resulted in an increase of the discussed coefficient to the value of 0.798.

Figure 18 shows the spectra of the lubricating compositions prepared using soybean oil without the additive and the composition modified with montmorillonite. In the spectrum, in addition to the peaks characteristic of the vegetable-based oil (3010 cm^−1^, 2896 cm^−1^, 1657 cm^−1^, 1441 cm^−1^), there were peaks characteristic of the Si-O bond (1302 cm^−1^, 1264 cm^−1^, 1079 cm^−1^) contained in the modifying additive, montmorillonite. The integral intensity of the characteristic bands of the vegetable-based oil (I_1441_/I_1657_) was calculated for the tested lubricating compositions. The analysis of the obtained data shows that the ratio of the relative intensity of characteristic bands increased after the introduction of the structure of montmorillonite, changing from 0.613 for the composition without modifier to 0.866 for the composition modified by montmorillonite.

Below in Table 7 is a summary of the calculated values of the integral intensity bands for the tested vegetable lubricant compositions.

Greases produced from vegetable oils (rapeseed, linseed and soybean) after the introduction of modifying agents in the form of Aerosil and Montmorillonite were studied using Raman spectroscopy to evaluate changes in the chemical structure of the vegetable-based oils. The analysis of the Raman spectra made it possible to study the changes occurring under the influence of the additives used in the range of multiple characteristic bands of unsaturated bonds and single saturated bonds located in the fatty acids of the vegetable-based oils. 

The application of Raman spectroscopy made it possible to evaluate the effect of the modifying additives on the changes in the structure of the vegetable oils used as the oil base of the tested lubricating compositions. It was observed that the introduction of amorphous-fumed silica and montmorillonite increased the integration intensity of the analysed bands I_1441_/I_1653_. Significant changes in the integral intensity bands indicate significant changes in the structure of the lubricating compositions under the influence of the additives used. The degree of unsaturation of fatty acids, determined on the basis of the intensity ratio I_1441_/I_1653_, increased in comparison with lubricating compositions not modified with silica additives, which indicates a change in the structural stability of the tested compositions, resulting in an improvement of their functional properties, which is of great applied importance.

The peaks show that after carrying out tribological tests on green lubricants with different types of modifiers, the presence of organometallic substances was detected on the outer film of the steel plate. The peaks at 3010 and 3008 cm^−1^ were associated with the hydrocarbon chain group involved in the formation of internal molecular hydrogen ions in the matrix of the base stocks used. The peaks at 2902, 2895, 2860 and 2856 cm^−1^ were the result of the superposition of peaks characteristic of symmetric and unsymmetric vibrations of the -CH_3_ and -CH_2_- groups originating from the hydrocarbon sequence of the oil bases of the greases studied. The region at 2730 cm^−1^ was caused by stretching vibrations of the methyl and methylene groups of the carbon sequence (-CH_2_-, -CH_3_-). In comparison, the peaks at 2090 and 2084 cm^−1^ were more related to stretching vibrations of the C=O group, typical of atmospheric decomposition products. The peak at 1752 cm^−1^ can be attributed to the C=O group motions characteristic of esters. The region at 1658 cm^−1^ is associated with the C=C group present in the hydrocarbon chain of the oil matrix. The peaks at 1444 and 1446 cm^−1^ corresponded to the -COO vibrations or the symmetrical and unsymmetrical transformations of the hydrocarbon group(s) contained in the carbon network of the crude oil and the additives used.

The peaks at 1310, 1304 and 1274 cm^−1^ are typical of skeletal vibrations of the -Si-O- group in layered silicate. The region at 1075–1079 cm^−1^ is representative of the structural changes of the -Si-O- group in the modified silica. The experiments carried out have shown that during tribological operations, some components of lubricants are subjected to oxidation. As a result, organo-oxides are formed in the film on the metallic contact surface, which counteract the wear of the lubricated sliding contact surface. These substances form an intimate bond with the top coat and increase its resistance to wear and friction. The effectiveness of the applied additives is achieved by the formation (during friction) of a highly resistant thin film, chemically bonded to the substrate, characterized by low shear forces, high plastic properties and excellent resistance to wear phenomena. 

As a result of the thermal degradation of the thickener and additives, chemical interactions take place between the constituents of the base component and the lubricating compositions. The advanced mixtures infiltrate the working areas of the tribological pair and form wear-protective surfaces. The interphase is formed by the conversion of lubricant elements into polar organic molecules and their subsequent reaction with metallic or oxide materials. The thermal and physical processes involved break down the carbon chains of the base oil and form new polar components. The end products of the frictional reactions are mixtures that form interfacial layers on the surface of the tribological system.

## 4. Conclusions

The determined welding load values showed that the use of hydrophilic amorphous silica and montmorillonite as modifying additives in vegetable lubricants effectively improves the anti-scuffing properties of the tested lubricating compositions with abruptly increasing loads on the tribo-system, but montmorillonite provides more effective anti-scuffing protection.

The scuffing limit pressure values have shown that the use of layered silicates in the form of montmorillonite has the best effect on improving the anti-scuffing properties of the lubricating compositions used in the experiment. The use of Aerosil-modified silica as a modifier improves the anti-stick properties in relation to the base grease compositions, but has a lower anti-stick effect than the phyllosilicates. 

The highest level of antiwear protection was achieved by using layered silicates, irrespective of the base oil used. The level of antiwear properties obtained makes all the tested lubricant compositions effective lubricants under a constant tribological load.

Under the influence of the applied modifiers, the G_oz_ parameter increases, which is evidenced by a high resistance to boundary layer breakage. This indicates a positive effect of the applied additives on the improvement of the tribological properties of vegetable lubricants.

For the composition based on vegetable oils, a positive effect of the applied nanoadditives in the form of amorphous silica and montmorillonite on the changes of the dynamic viscosity at high shear rates was observed. The viscosity value decreased in comparison with the basic composition without additives. The introduction of layered silicate in the form of montmorillonite into the structure of grease based on vegetable oils showed a negative effect of the used additive on viscosity changes at low shear rates, which was caused by an insufficient distribution of the used additive in the structure of the tested lubricant. The use of a nanoadditive containing silicon in lubricating greases based on rapeseed oil has a positive effect on the change in viscosity values at low shear rates. No significant changes in the dynamic viscosity at high shear rates were observed for greases produced using rapeseed oil. 

The results obtained allow us to state that the greases modified by ceramic additives are characterized by strange viscous properties compared to greases without additives, which could be caused by the change in the microstructure of the sample in question as a result of the processes of degradation of the oil base and the oxidation products formed.

The tests carried out on the vegetable greases modified with amorphous silica showed a decrease in the value of the G′ modulus in comparison with the results of the tests carried out on the basic vegetable greases. For greases modified with montmorillonite, a significant change in the value of the modulus was observed in the low frequency range, which is characteristic of the diffusion behavior in the dispersion system studied. When analyzing the loss modulus G″, significant differences in the value and shape of this modulus were observed for vegetable greases modified with ceramic additives in the high frequency range. It was observed that greases modified with silica and montmorillonite were characterised by much lower values than base greases.

At higher frequency values, the G′ modules were characterised by higher values. However, for greases based on linseed oil and modified of amorphous silica, the structure was changed, because the values of G″ modulus are much higher than the values of G′ modulus. This result proves the strong viscous properties of the tested greases. In the case of the montmorillonite-modified grease, the loss modulus (G″) is much higher than the storage modulus G′ in the low frequency range observed. This behavior of the greases tested is indicative of the stable viscosity in the frequency range tested.

As the dynamic viscosity decreases and as the flow limit and the magnitude of the thixotropic properties increase, characterised by the size of the hysteresis loop, the anti-stick and anti-wear properties of lubricants containing the amorphous silica Aerosil 300 and montmorillonite increase.

It was observed that the introduction of amorphous fumed silica and montmorillonite increased the integration intensity of the analyzed bands I_1441_/I_1653_. Significant changes in the intensity of the integral band indicate significant changes in the structure of lubricating compositions under the influence of the additives used. The degree of unsaturation of fatty acids, determined on the basis of the I_1441_/I_1653_ intensity ratio, increased in comparison with lubricating compositions not modified with silica additives, which indicates a change in the structural stability of the tested compositions, resulting in an improvement of their functional properties. This is of great importance for applications.

## Figures and Tables

**Table 1:** Properties of grease base oils.

Parameter to be Tested	Test Method	Unit	Rapeseed Base Oil	Linseed Base Oil	Soybean Base Oil
Kinematic viscosity at 40 °C	PN-EN ISO 3104:2021 [55]	mm^2^/s	36.24	31.12	31.87
Kinematic viscosity at 100 °C	PN-EN ISO 3104:2021 [55]	mm^2^/s	7.754	7.623	7.522
Viscosity index	PN ISO 2909:2009 [56]	-	192	206	217
Pour point	PN ISO 3016:2005 [57]	°C	−20	−19	−18
Flash point	PN-EN ISO 2719:2016-08 [58]	°C	282	286	292
Oxidation at 80 °C	ASTM D 942-19 [59]	h	34.53	38.43	32.43
Antiwear properties	WTWT-94/MPS-025 [60]	N/mm^2^	819.16	1007.64	754.62
Antiscuffing properties	PN-76/C-04147 [61]	N/mm^2^	422.04	575.12	529.78
Density at 20 °C	PN-EN ISO 12185:2002 [62]	g/cm^3^	0.862	0.902	0.822
Peroxide number	PN-EN ISO 3960:2017-03 [63]	meq O_2_/kg	2.66	2.88	2.44
Iodine number	PN-EN ISO 3961:2013-10 [64]	g I_2_/100 g	88.47	92.65	78.13
Acid number	PN-EN ISO 660:2010 [65]	mg KOH/g	0.26	0.39	0.31

**Table 2:** The chemical composition of the base oils used to make lubricating greases.

Base Oil	Saturated Fatty Acid Content [%]	Monounsaturated Fatty Acid Content [%]	Polyunsaturated Fatty Acid Content [%]
Rapeseed	7.4	63.3	28.1
Linseed	8.9	59.0	32.1
Soybean	15.7	22.8	57.7

**Table 3:** Physical and chemical properties of thickeners and additives for lubricating greases.

Parameter	Unit	Lithium Stearate (Thickener)	Montmorillonite (Additive)	Aerosil 300 (Additive)
Moisture content	%	1.23	5.50	1.50
Moh’s hardness	-	0.78	1.36	0.82
Melting point	°C	220	>1300	>1600
Compressive strength	RcwN/cm^2^	3.21	7.50	2.95
pH value	^-^	5.5	9.1	3.7–4.5
Color	-	white	white	white
Auto ignition Temperature	°C	211	190	220
Volatile content	%	0.04	0.06	0.12
Density at 20 °C	g/cm^3^	1.025	2.240	5.0
Flash point	°C	162	185	198
Refractive index	-	-	1.518	-
Boiling point	°C	359	378	394
Molar mass	g/mol	290.42	549.07	68.13
Chemical content				
O_2_	%	23.11	64.11	25.66
Na	%	0.02	0.84	0.04
Ca	%	0.03	0.73	0.08
Al	%	0.05	9.83	0.06
Si	%	0.08	20.46	74.12
H	%	26.77	4.04	0.03
C	%	47.54	-	-
Li	%	2.55	-	-

**Table 4:** Chemical composition of steel ball 100Cr6.

Chemical Content	Unit	Content in Steel Ball (100Cr6)
Fe	%	95.92–96.80
C	%	0.93–1.05
Si	%	0.15–0.35
Mn	%	0.25–0.45
P	%	Max. 0.025
S	%	Max. 0.015
Cr	%	1.35–1.60
Mo	%	Max. 0.10
Cu	%	Max. 0.30
Al	%	Max. 0.050
O	%	Max. 0.0015

**Table 5:** Parameters of rheological models describing the flow curves of basic and modified lubricating compositions with no additives.

(a) Bingham Model			
**Tested Grease**	**Coefficient of Determination R^2^ [** **-** **]**	**Yield Point τ_0_ [Pa]**	**Structural Viscosity η_∞_ [Pa·s]**
A	0.975	68.11	20.15
A1	0.911	404.37	18.85
A2	0.866	1234.55	3.45
B	0.982	431.44	16.14
B1	0.954	271.28	5.57
B2	0.978	593.84	75.78
C	0.905	316.30	7.48
C1	0.924	437.73	15.95
C2	0.888	368.92	33.61
(b) Casson model			
**Tested Grease**	**Coefficient of Determination R^2^ [** **-** **]**	**Yield Point τ_0_ [Pa]**	**Structural Viscosity η_∞_ [Pa·s]**
A	0.983	71.60	22.71
A1	0.921	416.43	21.44
A2	0.875	1263.92	4.50
B	0.991	452.73	17.92
B1	0.973	279.05	7.26
B2	0.989	606.65	82.10
C	0.913	327.08	8.85
C1	0.933	453.76	17.54
C2	0.878	386.13	35.15
(c) Herschel–Bulkley model			
**Tested Grease**	**Coefficient of Determination R^2^ [** **-** **]**	**Yield Point τ_0_ [Pa]**	**Structural Viscosity η_∞_ [Pa·s]**
A	0.988	74.28	26.76
A1	0.937	431.21	23.15
A2	0.904	1304.36	8.77
B	0.996	467.89	21.14
B1	0.981	298.12	9.91
B2	0.992	654.79	85.23
C	0.928	338.17	11.39
C1	0.946	469.34	21.49
C2	0.897	404.33	39.67
(d) Tscheuschner model			
**Tested Grease**	**Coefficient of Determination R^2^ [** **-** **]**	**Yield Point τ_0_ [Pa]**	**Structural Viscosity η_∞_ [Pa·s]**
A	0.986	72.46	24.11
A1	0.932	420.78	22.34
A2	0.893	1275.45	6.76
B	0.994	458.91	19.43
B1	0.978	286.33	8.54
B2	0.991	632.18	83.19
C	0.918	330.23	10.23
C1	0.938	458.56	19.88
C2	0.886	393.63	37.77

**Table 6:** The calculated value of the surface area of the loop hysteresis of the tested greases.

Tested Grease	Loop Hysteresis Surface Area Value [Pa]
A	198.77
A1	137.87
A2	573.66
B	88.38
B1	123.46
B2	764.63
C	68.75
C1	153.93
C2	978.54

**Table 7:** Comparison of the calculated values of the integral intensity band for greases produced using rapeseed, linseed and soybean dispersion phases with soap thickener and modified structures with Aerosil and montmorillonite.

Lubricating Greases	Raman Spectroscopy—Integral Intensity Bands I_1657_/I_1441_	Raman Spectroscopy—Integral Intensity Bands % Change after Use of Modifying Additive
Grease A	0.530	-
Grease A1	0.672	+26.79
Grease A2	0.842	+58.87
Grease B	0.582	-
Grease B1	0.756	+29.90
Grease B2	0.889	+52.75
Grease C	0.613	-
Grease C1	0.798	+30.18
Grease C2	0.866	+41.27

## Data Availability

All data are available upon request.
